# Targeting inflammation in cardiometabolic disease: Icosapent ethyl modulates monocyte‐derived macrophages isolated from patients with cardiovascular disease with or without type 2 diabetes

**DOI:** 10.1111/dme.70247

**Published:** 2026-02-09

**Authors:** J. K. Ward, M. U. Shah, K. Lee, P. E. Squires, C. E. Hills

**Affiliations:** ^1^ Cardiorenal Team, Diabetes, Metabolism & Inflammation Group, College of Health and Science, Joseph Banks Laboratories University of Lincoln Lincoln UK; ^2^ Lincolnshire Heart Centre United Lincolnshire Hospitals Lincoln UK; ^3^ Cardiology Department Hull University Teaching Hospitals Hull UK

**Keywords:** icosapent ethyl, inflammation, macrophages, NLRP3 inflammasome, senescence

## Abstract

**Aims:**

Despite intensive lipid‐lowering therapy, individuals with atherosclerotic cardiovascular disease (ASCVD) exhibit residual inflammatory risk, which drives recurrent cardiovascular events. This risk is amplified in type 2 diabetes mellitus (T2DM), where a pro‐inflammatory milieu accelerates atherogenesis. Monocyte‐derived macrophages (MDMs), key mediators of vascular inflammation, contribute significantly to this process. Icosapent ethyl (IPE), a highly purified ethyl ester of eicosapentaenoic acid (EPA), reduces major adverse cardiovascular events (MACE) beyond triglyceride lowering, yet its cellular mechanisms remain unclear. This study aims to determine whether IPE modulates inflammatory pathways in patient‐derived MDMs and to distinguish direct EPA effects from therapy‐mediated changes.

**Methods:**

This single‐centre, open‐label, randomised observational cohort study will recruit ASCVD patients, stratified by T2DM status, who are prescribed IPE (Vazkepa®). MDMs and plasma/serum samples will be collected from patients, either IPE‐naïve or following 6 months of therapy. In parallel, direct EPA effects will be assessed by treating MDMs from healthy donors and ASCVD patients with physiologically relevant concentrations of EPA. We will evaluate NOD‐like receptor protein 3 (NLRP3) inflammasome priming and activation, inflammatory cytokine profiles, and markers of cellular senescence.

**Results:**

The study will investigate mechanisms that potentially underlie the cardiovascular benefits of IPE, focusing on the modulation of inflammatory pathways. We hypothesise that IPE attenuates priming and activation of the NLRP3 inflammasome in monocyte‐derived macrophages, thereby reducing cellular inflammation and senescence.

**Conclusions:**

This study will provide mechanistic insight into how IPE influences macrophage‐driven inflammation in ASCVD and T2DM, informing strategies to target residual inflammatory risk in high‐risk cardiometabolic populations.

## 1 INTRODUCTION

Atherosclerotic cardiovascular disease (ASCVD), a chronic inflammatory disorder, is the principal pathology underlying cardiovascular disease (CVD), the leading global cause of morbidity and mortality.^1^ Individuals with type 2 diabetes (T2DM) face a markedly increased risk of ASCVD, with coronary artery disease accounting for at least 50% of deaths in this high‐risk population.^2^ Its progression is primarily driven by dyslipidaemia, characterised by elevated low‐density lipoprotein cholesterol (LDL‐C), triglycerides and other lipid abnormalities. Individuals with T2DM exhibit a higher prevalence of these lipid disturbances, further elevating ASCVD risk. This dyslipidaemia is often compounded by comorbidities such as obesity and genetic or ethnic predispositions.^3^ Although statins remain the cornerstone of lipid management, many patients continue to experience persistent hypertriglyceridaemia despite optimal therapy.^4^ This residual risk highlights the need for adjunct treatments to address the complex interplay between dyslipidaemia, chronic inflammation and cardiometabolic disease.^5^, ^6^

Icosapent ethyl (IPE), a highly purified and stable ethyl ester of eicosapentaenoic acid (EPA), is indicated for high‐risk patients with established ASCVD who have residual hypertriglyceridaemia (>1.7 mmol/L) despite statin therapy. The Reduction of Cardiovascular Events with Icosapent Ethyl–Intervention Trial (REDUCE‐IT) (NCT01492361) demonstrated that IPE significantly reduced major adverse cardiovascular events (MACE) in patients with ASCVD or T2DM plus additional risk factors.^7^ Interestingly, clinical benefits were observed even among patients with normal baseline triglycerides, suggesting a mechanism of action beyond lipid lowering.^8^ Reductions in circulating inflammatory biomarkers, including high‐sensitivity C‐reactive protein (hsCRP), have been reported^9^; however, the precise cellular mechanisms by which IPE modulates inflammation and reduces cardiovascular risk remain unclear. Studies to date have predominantly focused on plasma biomarkers^10^ or in vitro models,^11^, ^12^, ^13^ with limited investigation on patient‐derived cells. This leaves a critical knowledge gap in our understanding of how IPE influences vascular inflammation, notably the behaviour of immune cells, at the cellular and molecular level.

In ASCVD, immune cell activation is a central driver of vascular inflammation. Elevated circulating cluster of differentiation (CD)14^+^ monocytes and tissue‐infiltrating C‐C chemokine receptor type‐2 (CCR2^+^) monocyte‐derived macrophages (MDMs) are associated with adverse cardiovascular outcomes,^14^, ^15^ while activation of the NOD‐like receptor protein‐3 (NLRP3)‐inflammasome contributes to endothelial dysfunction,^16^ plaque progression and increased cardiovascular risk.^17^ Furthermore, blood‐borne CD14^+^ MDMs retain a trained immuno‐metabolic memory, enabling enhanced or suppressed responsiveness upon re‐stimulation.^18^ Given their long lifespan in the arterial wall, which can range from months to years, MDMs represent a critical cellular driver of chronic vascular inflammation and an ideal ex vivo model for assessing immune responses pre‐ and post‐intervention.

Clinical evidence demonstrates that inhibition of the NLRP3 inflammasome/interleukin (IL)‐1β/IL6 pathway reduces non‐fatal cardiovascular events^19^ and biomarkers relevant to atherosclerosis.^20^ Activation of this pathway triggers a cascade of events, including cellular senescence, macrophage polarisation and impaired autophagy, each of which independently contributes to adverse cardiovascular outcomes.^21^ Given their cumulative impact, targeting the NLRP3/IL1β/IL6 axis represents a clinically important strategy for addressing residual inflammatory risk in patients with cardiovascular disease.

This study will assess the impact of IPE therapy on NLRP3‐inflammasome activation, inflammatory signalling and immuno‐senescence in CD14^+^ MDMs isolated from individuals with ASCVD, with or without T2DM, who are either IPE‐naive or have received 6 months of therapy. Given that clinical benefits of IPE have been observed even in patients with normal baseline triglycerides, we will also examine the direct effects of EPA on our candidate pathways of interest and other markers of inflammation in MDMs derived from healthy donors and from patients with ASCVD ± T2DM (IPE‐naïve). Collectively, data from this study will help distinguish the direct cellular effects of EPA from indirect, therapy‐mediated changes observed in patients.

## 2 METHODS/DESIGN

### 2.1 Study type

This is a single‐centre, prospective, open‐labelled, observational cohort study with randomisation to immediate or delayed initiation of IPE, without a placebo control—ISRCTN64669648.

### 2.2 Primary outcome

Change in NLRP3‐inflammasome activation in CD14^+^ MDMs: Assessed in individuals with ASCVD ± T2DM following 6 months of IPE therapy compared with IPE‐naïve controls. This will include quantification of NLRP3 complex assembly, caspase‐1 activation and downstream IL1β production.

### 2.3 Secondary outcomes

Inflammatory signalling profile in patient‐derived MDMs: Evaluation of key inflammatory pathways linked to ASCVD progression, including IL6/tumour necrosis factor‐alpha (TNFα) signalling and macrophage polarisation.

Immuno‐senescence phenotype in CD14^+^ MDMs: Assessment of senescence‐associated markers (e.g. p16, p21, BCL2) and senescence‐associated secretory phenotype (SASP) components (IL8, CCL2).

### 2.4 Direct effects of EPA on immune‐cell inflammatory responses (ex‐vivo arm)

Using MDMs derived from healthy donors and ASCVD ± T2DM patients (IPE‐naïve), we will determine how purified EPA modulates NLRP3 activation, cytokine secretion, macrophage programming and other inflammatory and immuno‐senescence‐associated pathways. These experiments will help discriminate the direct cellular actions of EPA from indirect effects that may arise in vivo during IPE therapy.

### 2.5 Recruitment

**FIGURE 1:**
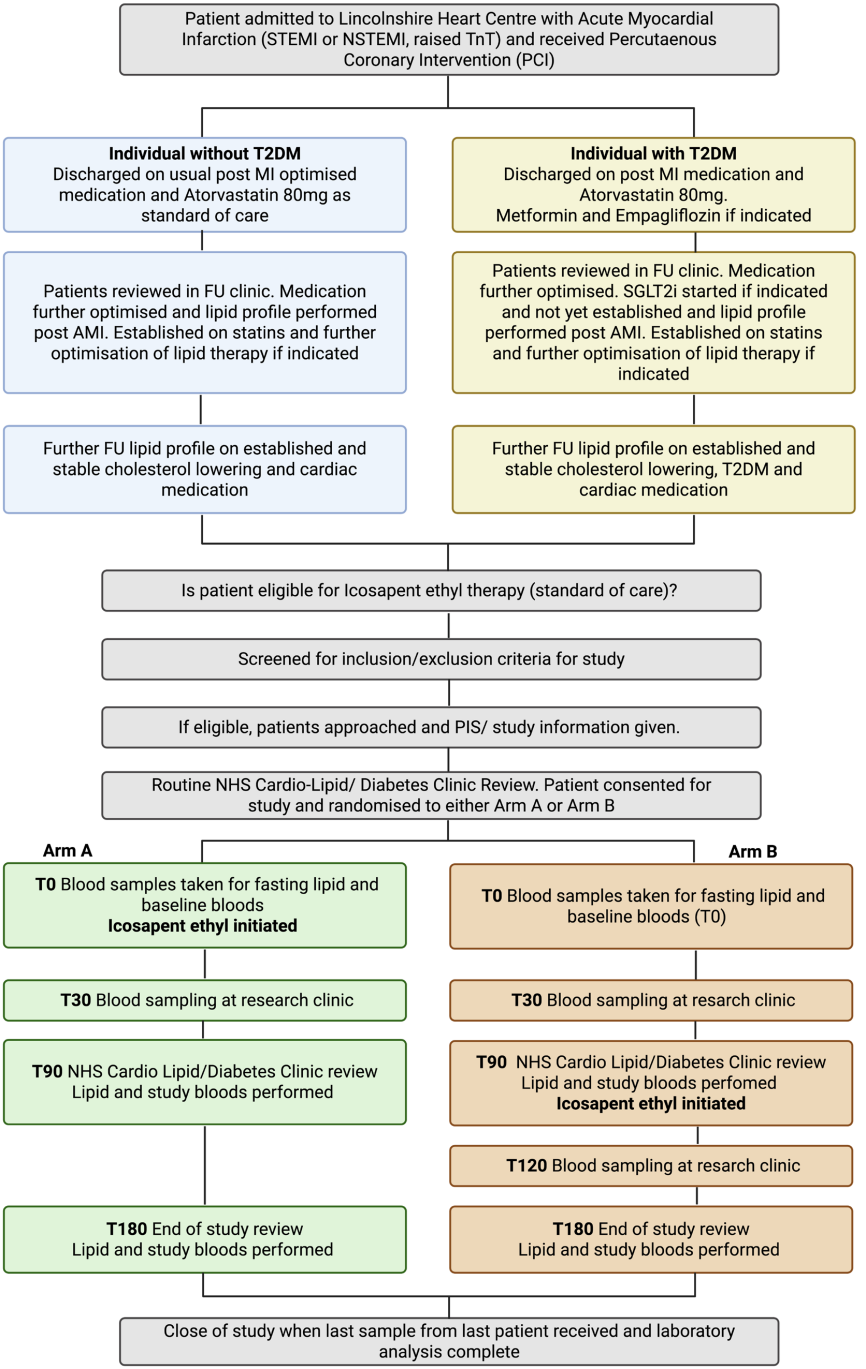
Flowchart demonstrating patient selection, patient consent, randomisation and standard of care/research clinic appointments. FU, follow‐up; NSTEMI, non‐ST elevation myocardial infarction; PIS, participant information sheet; STEMI, ST elevation myocardial infarction; TnT, troponin T.

**FIGURE 2:**
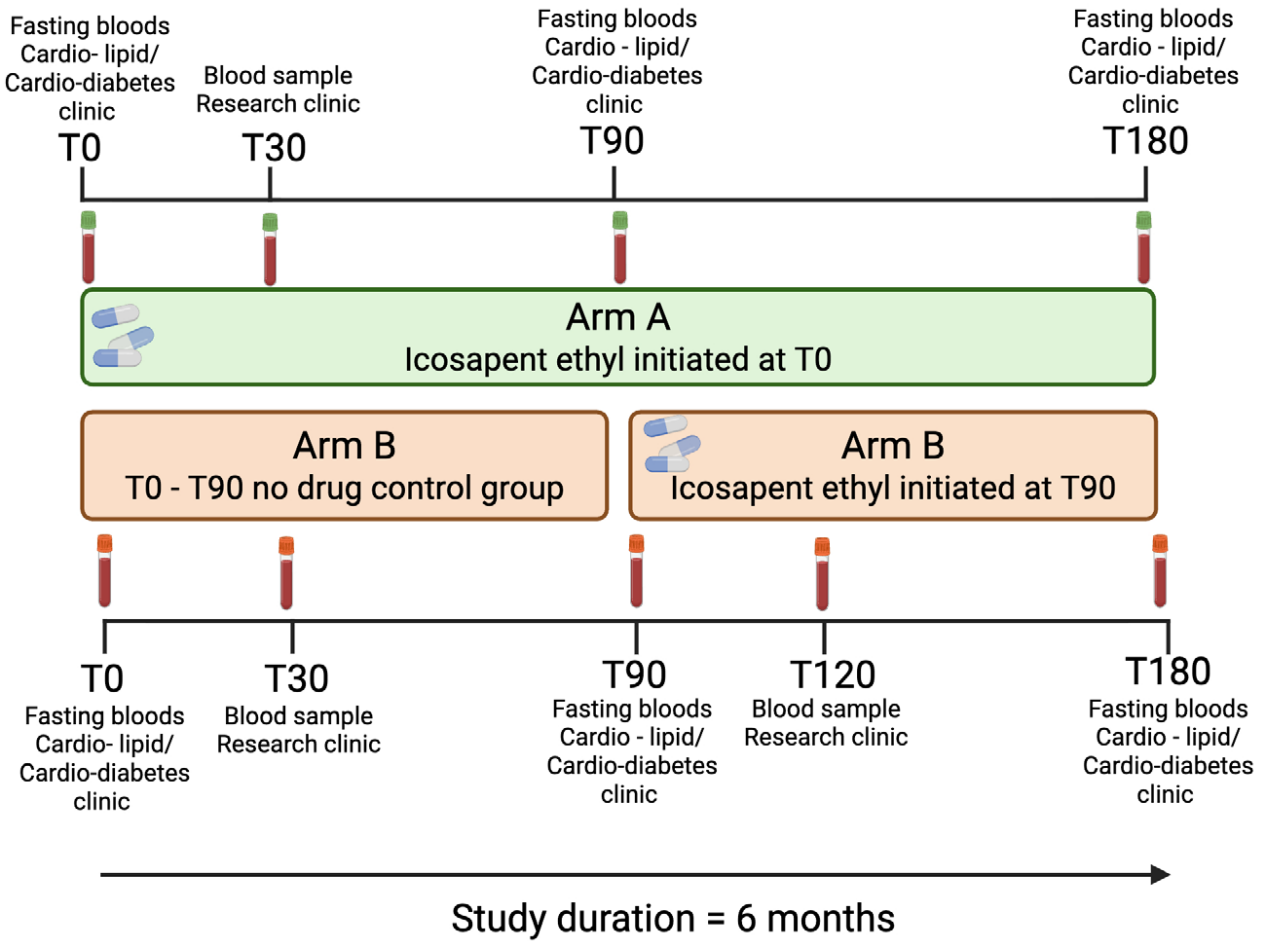
Schematic demonstrating the timing of initiation for IPE in Arm‐A and Arm‐B.

**FIGURE 3:**
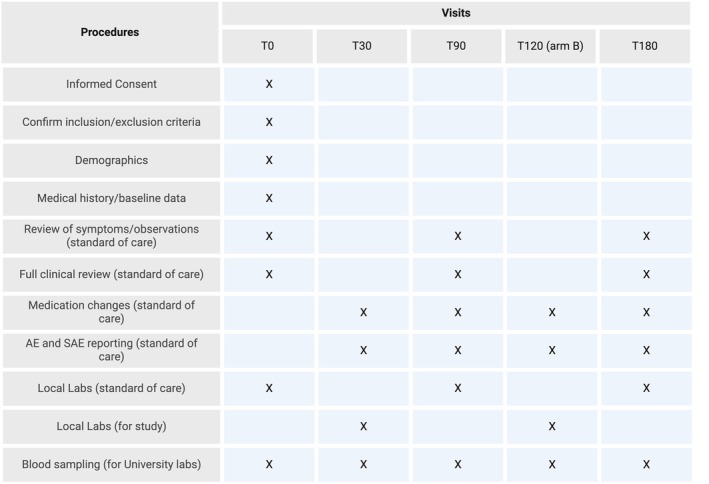
Schedule of procedures for randomisation and follow‐up visits. AE, adverse event; SAE, serious adverse event.

IPE Study: We will recruit sixty‐six participants (*n* = 30 per arm, with a 10% contingency in the event of dropout), eligible for IPE therapy based on their usual standard of care (Figure 1). Participants will be randomised to one of two arms to receive IPE either on day 0 (Arm‐A) or 90 days after enrolment in the study (Arm‐B) (Figure 2), with an equal distribution of individuals with and without T2DM per arm (*n* = 33 each). Participation will be approximately 6 months from consent to the final visit and will involve sampling at T0, T30, T90, T180 (Arm‐A) or T0, T30, T90, T120 and T180 (Arm‐B) (Figure 3).

Sample size was based on detecting a biologically meaningful 25% reduction in NLRP3‐inflammasome activation in CD14^+^ MDMs following 6‐month IPE therapy compared with delayed treatment. Prior in‐house and published evidence on inflammasome and cytokine assays in similar patient populations indicates a within‐group coefficient of variation of approximately 30%–35%. Assuming a two‐sample, two‐sided *t*‐test with *α* = 0.05 and 80% power, an effect size (Cohen's *d*) of approximately 0.7 requires about 32 participants per group (*n* = 64). For feasibility, we set a base of 30 participants per arm and increased this by 10% to allow for anticipated dropout, giving a recruitment target of 66 participants (33/arm). This sample size provides adequate power to detect clinically meaningful changes in NLRP3 activation and related inflammatory markers.

EPA work: For the assessment of the ‘direct’ in‐vitro effects of EPA on healthy donor‐derived MDMs, the sample size was estimated based on detecting a biologically meaningful 20%–25% change in NLRP3‐inflammasome activation or downstream cytokine release in CD14^+^ MDMs following exposure to EPA. Prior in‐house variability estimates and published data for primary human monocyte/macrophage assays indicate a within‐donor coefficient of variation of approximately 20%–30%. Under a paired design, with each donor serving as their own control, this variability supports the detection of moderate effect sizes (Cohen's *d* ≈ 0.8) with 80% power at *α* = 0.05 using 10 donors per group. Accordingly, we will recruit 10 healthy donors, which is sufficient to detect EPA‐mediated alterations in inflammasome activity and inflammatory signalling while enabling comparison between healthy and disease‐derived immune cells. Healthy donors will be eligible if they have a BMI of 18.5–24.9, no chronic or acute inflammatory conditions and are not taking medications with anti‐inflammatory effects (e.g. non‐steroidal anti‐inflammatory drugs).

Similarly, MDMs isolated from IPE naïve patients (*n* = 20) will be used to study the direct effects of EPA on NLRP3 inflammasome activation and downstream inflammatory cytokine secretion to assess the direct effects of EPA in diseased cells, in addition to comparing the response observed in healthy cells.

### 2.6 Data collection and statistical analysis

IPE study: Data will be pseudo‐anonymised and will include clinical data, patient demographics and parameters of interest. To assess both primary (NLRP3 inflammasome priming and activation) and secondary outcomes (ATP release, cytokine secretion and markers of immunosenescence), a paired *t*‐test (parametric data) or a Wilcoxon matched pairs signed‐rank test (non‐parametric data) will determine intra‐arm changes in our markers of interest at T0 versus T30 and T0 versus T90 in MDMs isolated from patients in Arm A (IPE) and Arm B (IPE naïve). For inter‐ and intra‐arm analysis of percentage change from baseline, measurements will use a two‐way ANOVA with Tukey's post‐test. A *p* < 0.05 will be considered statistically significant. Statistical analysis will be carried out using GraphPad Prism.

EPA work: In the first instance, an unpaired *t*‐test with Welch's correction (parametric data) or a Mann–Whitney test (non‐parametric data) will compare baseline levels of our markers of inflammation (NLRP3 inflammasome activation, ATP release, cytokine secretion and immunosenescence) in MDMs isolated from healthy donors versus individuals with ASCVD (IPE naïve).

For the assessment of the ‘direct’ effect of EPA (multiple treatment conditions) on parameters of interest (NLRP3 inflammasome activation, ATP release, cytokine secretion and immunosenescence) in LPS‐stimulated MDMs isolated from each group, a one‐way ANOVA with Tukey's post‐test (parametric data) or a Kruskal–Wallis with Dunn's post‐test will be used. A *p* < 0.05 will be considered statistically significant. Statistical analysis will be carried out using GraphPad Prism.

### 2.7 Eligibility

Inclusion criteria:

- Patients with established ASCVD, defined as documented evidence of one or more of the following, including a prior event of acute myocardial infarction (AMI) or previous revascularisation (by percutaneous coronary intervention [PCI] or coronary artery bypass graft surgery [CABG]).
- With or without a known T2DM as confirmed through medical record review, including a documented clinical diagnosis of T2DM, concurrent treatment with glucose‐lowering therapies, or historical laboratory evidence meeting diagnostic criteria such as HbA_1c_ ≥48 mmol/mol on at least 2 occasions or on one occasion in the presence of typical symptoms of diabetes as per World Health Organisation criteria.
- If T2DM, the disease must be established and clinically stable on oral glucose‐lowering medications.
- Established and stable on statin and/or ezetimibe therapy.
- LDL‐C levels above 1.04 mmol/L and below or equal to 2.60 mmol/L in line with NICE eligibility for IPE therapy at the time of recruitment.
- Raised fasting triglycerides (1.7 mmol/L or above).
- Eligible for IPE therapy within licensed indication and National Institute for Health and Care Excellence (NICE) guideline criteria.
- Able to provide informed consent.

Exclusion criteria:

- Contraindications to IPE therapy, including known hypersensitivity to IPE, EPA, fish or shellfish.
- Concurrent or planned bempedoic acid, inclisiran or PCSK9 (proprotein convertase subtilisin/kexin type‐9) therapy.
- Pregnancy or breast feeding.
- Severe end‐stage kidney failure, defined as an estimated glomerular filtration rate (eGFR) <15 mL/min/1.73 m^2^ or requirement for chronic dialysis.
- Severe end‐stage liver disease, defined as decompensated cirrhosis or clinical features of liver failure, including ascites, hepatic encephalopathy or variceal bleeding.
- Other conditions that would reduce the expected life span of a patient to less than 2 years.
- Unable to provide informed consent.
- Acute renal failure or acute kidney injury, defined biochemically as a rapid increase in creatinine to more than 1.5 times the baseline with a corresponding drop in estimated eGFR, known or presumed to have occurred within the previous 7 days, or an increase of 26 μmol/L or more within 48 h, as per Kidney Disease: Improving Global Outcomes (KDIGO) criteria.^22^
- Cardiogenic shock.
- Severe valvular heart disease.
- Recent acute cardiac event, revascularisation or surgery (within 3 months).
- Recent infective event (within 3 months).
- Active inflammatory‐related conditions, including infection, cancer or autoimmune disease.

Female participants will be assessed for childbearing potential. Non‐childbearing potential is defined as women who are either permanently sterilised (bilateral salpingectomy/oophorectomy or hysterectomy) or if they are post‐menopausal that is, amenorrhoeic (cessation of monthly period), for >12 months following cessation of exogenous hormone therapy and either:

- Aged >50 years, or
- Aged <50 years and have follicle‐stimulating hormone levels in the postmenopausal range.

Participants who do not meet these criteria will be offered a urine pregnancy test, as per clinical assessment, to rule out pregnancy. Women using effective contraception, including the combined oral contraceptive pill, will be considered women of childbearing potential. Routine contraception or abstinence will not be mandated, as IPE is prescribed as part of standard clinical care. If pregnancy is identified at screening or during the study, IPE therapy will be discontinued immediately, the participant will be withdrawn from further study procedures and no additional samples will be collected. The pregnancy will be managed according to local clinical procedures.

### 2.8 Blood sampling

Approximately 35–40 mL of blood will be obtained from individuals participating in the study. Patient samples will be transferred from Lincoln County Hospital to the University of Lincoln via an approved cold chain process. Blood samples (30 mL) from healthy donors will be taken with informed consent at Joseph Banks Laboratories (University of Lincoln—Lincoln Ethics Application System [LEAS] UoL2023_15828).

### 2.9 Blood sample preparation

Whole blood samples will be diluted in 1x phosphate‐buffered saline prior to density gradient centrifugation. Plasma will be retained from the peripheral blood mononuclear cell (PBMC) isolation with serum obtained from the centrifugation of blood in serum separator tubes, with both components decellularised and stored at −80°C. CD14^+^ monocytes will be isolated using CD14+ magnetic beads (Miltenyi, Germany) and differentiated into macrophages using macrophage colony‐stimulating factor over 6 days, with media replaced after 3 days.

### 2.10 Sample analysis

Activation of the NLRP3 inflammasome occurs through a two‐step process involving NFκB‐mediated IL1β priming followed by caspase‐1 dependent cleavage of pro‐IL1β and pro‐IL18, with ATP‐dependent P2X7 receptor activation serving as a key trigger. This study will assess whether NLRP3 priming, activation, and downstream markers of inflammation, macrophage inflammatory status and cell senescence are reduced in lipopolysaccharide (LPS)‐stimulated patient‐derived MDMs following IPE therapy.

**FIGURE 4:**
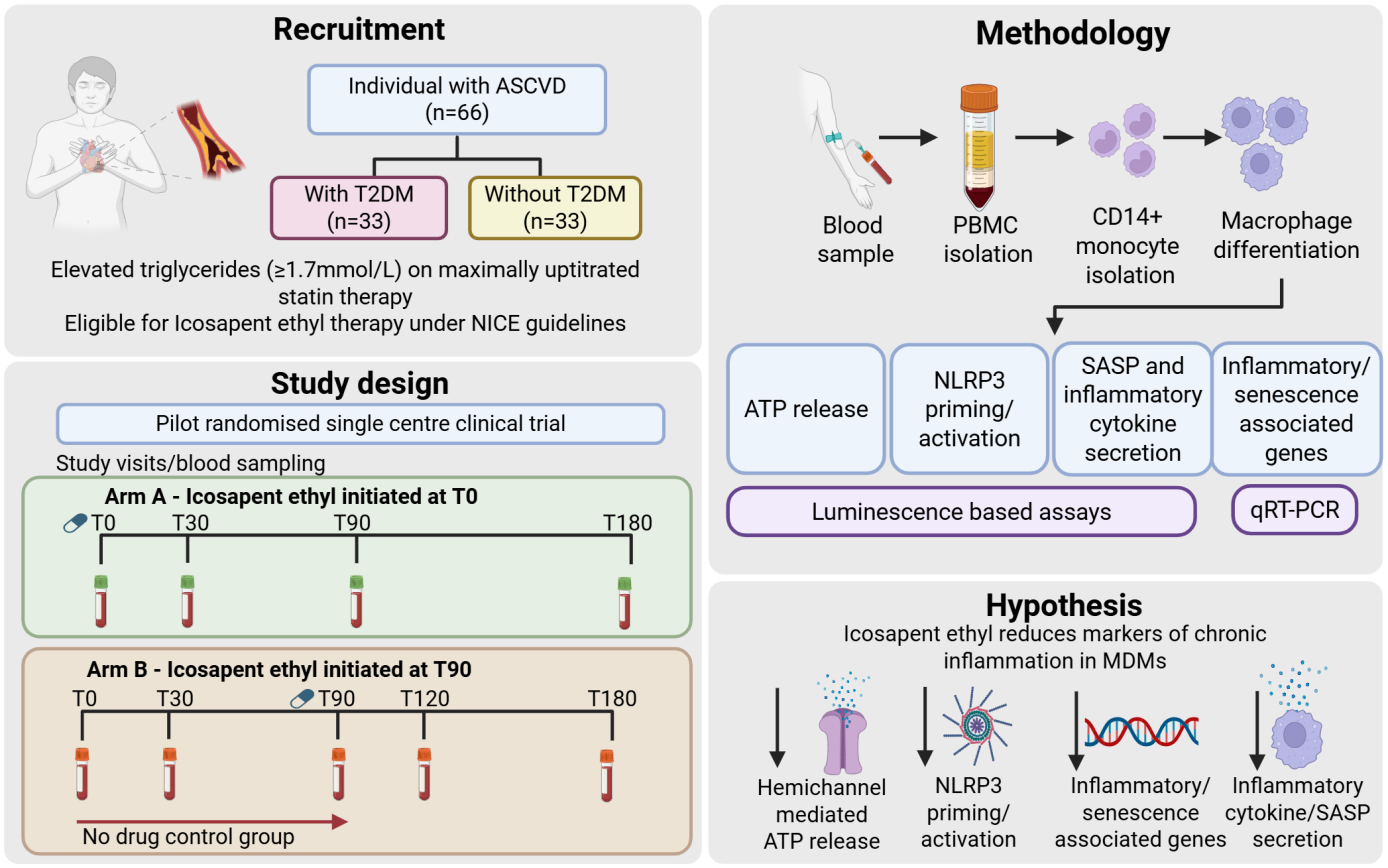
Study design and methodology for investigating the anti‐inflammatory effects of IPE in MDMs isolated from individuals with cardiovascular disease +/− T2DM. ASCVD, atherosclerotic cardiovascular disease; ATP, adenosine triphosphate; CD14, cluster of differentiation 14; IPE, icosapent ethyl; NICE, National Institute for Health and Care Excellence; NLRP3, Nod‐like receptor protein 3; PMBC, peripheral blood mononuclear cells; qRT‐PCR, quantitative real‐time polymerase chain reaction; SASP, senescence‐associated secretory phenotype.

Experiments will be conducted throughout the six‐month study. Inter‐ and intra‐arm analyses will evaluate changes within and between groups, enabling assessment of IPE temporal effects (Arm‐A, T0–T180) and comparative efficacy (Arm‐A T0–T90 vs. Arm‐B T0–T90) (Figure 4). Equal recruitment of participants with and without T2DM will allow correlation of treatment effects with diabetes status.

### 2.11 Caspase‐1 inflammasome Glo® assay

The effect of IPE on caspase‐1 activation will be assessed using the Caspase‐1 Inflammasome Glo® assay (Promega, US). MDMs will be treated with LPS and ATP prior to the addition of the lyophilised substrate solution as per manufacturer instructions. Pre‐incubation of MDMs with caspase‐1 inhibitor YVAD‐CMK or NLRP3 inhibitor MCC950 will confirm specificity. For the assessment of the direct effects of EPA, MDMs will be pre‐incubated with EPA for 24 h.

### 2.12 ATP lite luminescence assay

Extracellular ATP release will be quantified using the ATP lite luminescence assay (Revvity, US) in LPS stimulated MDMs pre‐treated with anti‐ectonucleotidase. Luminescence will be recorded after the addition of lyophilised substrate solution as per the manufacturer's instructions. Hemichannel blockers will be used in T0 samples to confirm the hemichannel‐mediated nature of the ATP release. For the assessment of the direct effects of EPA, MDMs will be pre‐incubated with EPA for 24 h.

### 2.13 RNA isolation and cDNA preparation

Total RNA will be isolated using TRIzol reagent (Invitrogen, USA), followed by chloroform‐isopropanol extraction, and reverse‐transcribed into cDNA with a High‐Capacity cDNA Reverse Transcription Kit (Applied Biosystems, USA) as per the manufacturer's instructions.

### 2.14 Quantitative real‐time polymerase chain reaction (qRT‐PCR)

Performed using qPCRBIO SyGreen Blue Mix with ROX dye (PCR Biosystems, UK) and specific oligonucleotide primers to quantify mRNA expression of the selected targets (Table S1). We will determine the expression of IL1β (NLRP3 priming), M1‐like macrophage markers (TNFα, IL6, signal transducer and activator of transcription 1 (STAT1), cluster of differentiation (CD)80), M2‐like macrophage markers (CD36, IL10), markers of immunosenescence (p16, p21, B‐cell lymphoma 2 [BCL‐2], BCL‐2 associated X [BAX], monocyte chemoattractant protein 1 [MCP1]) and membrane channel proteins (connexin 43 [Cx43] and pannexin‐1 [Panx1]).

### 2.15 Measurement of IL1β, TNFα, IL6 and IL10 release from MDMs

Human Lumit immunoassays (Promega, US) will measure the secretion of IL1β in LPS and ATP‐stimulated cells, in addition to measuring TNFα, IL6, IL8 and IL10 release in LPS‐stimulated cells. For the assessment of the direct effects of EPA, MDMs will be pre‐incubated with EPA for 24 h.

### 2.16 Proteome profiler human XL cytokine array

The array will be performed following the manufacturer's instructions. Briefly, cell supernatant from LPS ± EPA‐treated MDMs will be incubated overnight with pre‐blocked membranes spotted in duplicate with capture antibodies. Detection of antibodies will be performed using streptavidin and biotinylated antibody cocktails and visualised using chemiluminescence reagents. Image capture will be done via a Licor Odyssey FC imager on the chemiluminescence setting for 1 h. Pixel density for semi‐quantification of cytokine secretion will be performed using Licor Image Studio software v5.2.5.

### 2.17 Western blotting

Whole cell lysates will be prepared and separated via SDS‐PAGE gel electrophoresis and transferred onto Immobilon‐FL PVDF membranes as previously described.^23^ Membranes will be blocked for 1 h at RT with Intercept blocking buffer (Licor), then probed overnight at 4°C with specific polyclonal antibodies against markers of interest such as Cx43, p21, High‐Mobility Group Box 1 (HMGB1) or housekeeping protein α‐Tubulin (1:20,000). After four 5 ‐min washes with PBS‐Tween (0.01%), membranes will be probed with corresponding anti‐rabbit or anti‐mouse secondary antibodies (1:20,000) at RT for 1 h. Bands will be visualised using an Odyssey FC imaging unit and semi‐quantified using ImageStudio software (v5.2, Licor).

### 2.18 Safety

IPE is licensed for the treatment of individuals with established ASCVD and hypertriglyceridaemia (fasting triglycerides >1.7 mmol/L). Eligible individuals will be commenced on IPE therapy as per the recommended standard of care.

### 2.19 Withdrawal

Participants may withdraw from the study at any time, with no effect on their medical rights or standard of care. All data and samples collected up to the point of withdrawal will be retained and included in the analysis unless the participant explicitly requests the removal of their data. No further data or samples will be collected following withdrawal.

### 2.20 Study management

This study is registered under the ISCRTN (ISRCTN64669648), with ethical approval from the East of Scotland Research Ethics service (NHS REC:24/ES/006) and the Health Research Authority (335916).

## 3 DISCUSSION

Targeting residual cardiovascular risk beyond traditional lipid‐lowering therapies represents an important therapeutic strategy in the management of ASCVD and its complications. Recent evidence indicates that residual inflammatory risk persists despite optimal lipid control and contributes significantly to MACE in patients undergoing PCI.^24^ Understanding the cellular and molecular pathways that underpin inflammation in ASCVD, remains an unmet clinical need.

Post hoc analyses of REDUCE‐IT demonstrated that the cardioprotective effect of IPE on MACE and cardiovascular mortality extends beyond its triglyceride‐lowering activity and is independent of LDL‐C control.^25^ Patients with higher circulating EPA experienced the greatest clinical benefit, rather than those achieving the largest reductions in triglycerides.^26^ These findings suggest that IPE confers additional cardiovascular protection through mechanisms unrelated to lipid lowering, warranting further mechanistic investigation.

CD14^+^ monocytes and MDMs are central to ASCVD, with elevated circulating levels associated with adverse cardiovascular outcomes, including non‐fatal myocardial infarction and cardiovascular death.^10^ Modulating the pro‐inflammatory behaviour of these cells may represent a key mechanism through which IPE exerts cardiovascular benefit. Extensive in vitro and in vivo evidence supports an anti‐inflammatory role for EPA across diverse cell types, including endothelial cells and MDMs.^11^, ^12^, ^13^ However, these studies have primarily focused on EPA using a combination of murine models and immortalised MDMs. To date, no studies have explored how IPE influences the inflammatory phenotype of MDMs isolated from patients with ASCVD, with or without T2DM.

This study will determine the effect of IPE when prescribed to individuals with ASCVD +/− T2DM on macrophage inflammation. Our primary outcome will assess NLRP3‐inflammasome priming and activation, whilst the use of EPA on healthy donor and patient (Vazkepa naive)‐derived MDMs will help delineate the direct versus indirect effect of EPA on our observed outcomes. Additionally, we will assess the impact on downstream inflammatory pathways with an established link to residual inflammatory risk in T2DM and CVD. The beneficial outcomes observed in CANTOS (NCT01327846)^19^ demonstrated that antagonising components of the NLRP3/IL1β pathway improved cardiovascular outcomes and may provide a plausible mechanistic link. Furthermore, evidence points to the beneficial effects of EPA on the NLRP3 inflammasome.^27^, ^28^ To evaluate the effect of icosapent ethyl on NLRP3 activity in patient‐derived MDMs, we will quantify IL1β mRNA (as a marker of inflammasome priming), along with caspase‐1 activity and IL1β secretion. Moreover, with aberrant hemichannel‐mediated ATP release linked to P2X7R‐NLRP3 activation across multiple conditions of inflammation and ageing,^29^, ^30^ we will assess if IPE reduces inflammasome activation and downstream inflammatory signalling by inhibiting the release of this danger‐associated molecular pattern (DAMP). The contribution of connexin and pannexin hemichannels to this response will be assessed using established hemichannel blockers, for example Peptide 5^28^ and by measuring mRNA expression of Cx43 and Panx1 across the time course of the study.

In CVD, heightened NLRP3‐inflammasome activation is linked to the preferential polarisation of M1 macrophages and the induction of downstream cell senescence, events that exacerbate plaque instability, inflammation and ultimately contribute to ASCVD progression.^31^ We will measure mRNA expression of markers of senescence (p21, BCL‐2, BAX), macrophage inflammatory status (STAT1, CD80, CD36, IL10, TNFα) and secretion of the pro‐inflammatory SASP (IL6, TNFα, MCP‐1) to determine whether IPE mitigates senescence‐associated inflammation.

### 3.1 Limitations

The open‐label design and absence of a placebo control represent inherent weaknesses and may introduce bias. However, a placebo‐controlled design was not considered ethically appropriate, as icosapent ethyl is prescribed as part of standard clinical care for eligible patients in accordance with NICE guidance.^32^ To mitigate these limitations, we employed a delayed‐treatment randomisation strategy, allowing intra‐ and inter‐arm comparisons over predefined timepoints. In addition, the primary and secondary endpoints are objective laboratory‐based cellular and molecular measures, which are less susceptible to expectation or reporting bias.

### 3.2 Conclusions

Focusing on the regulation of immune cell behaviour, this study will characterise the effects of IPE on patient‐derived MDMs to identify cellular mechanisms that underpin its cardiovascular benefits in patients with hypertryglyceridaemia with or without T2D.

## FUNDING INFORMATION

This work is supported by Amarin Global and the University of Lincoln.

## CONFLICT OF INTEREST STATEMENT

None.

## Supporting information


Table S1.

## Data Availability

Data sharing not applicable to this article as no datasets were generated or analysed during the current study.
