# Supplementary material for: Targeting inflammation in cardiometabolic disease: Icosapent ethyl modulates monocyte‐derived macrophages isolated from patients with cardiovascular disease with or without type 2 diabetes
Source: Diabet Med. 2026 Feb 9;43(4):e70247. doi: 10.1111/dme.70247 (PMC12982656; doi:10.1111/dme.70247)
Supplement: Supplementary file 1 — Table S1. [file DME-43-e70247-s001.docx]

| Gene | Forward | Reverse |
| --- | --- | --- |
| 18s | 5'GTTGGTTTTCGGAACTGAGG3' | 5'GCATCGTTATGGTCGGAAC3' |
| BAX | 5'ATGGACGGGTCCGGGGAG3' | 5'ATCCAGCCCAACAGCCGC3' |
| BCL2 | 5'AACTGTACGGCCCCAGCAT3' | 5'GCCAAACTGAGCAGAGTCTTCAG3’ |
| CD36 | 5'CAGGAATGGAGACGCCCACA3' | 5'GGAGCGCTAGGGTTTACGGT3' |
| CD80 | 5'GGGAAATGTCGCCTCTCTGAAG3' | 5'ATTGGAGGGTGTTCCTGGGTC3' |
| Cx43 | 5'TGAGCCAGGTACAAGAGTGTGG3' | 5'GGAGATGAGCAGTCTGCCTTTC3' |
| IL1β | 5'TGGCAGAAGTACCTGAGCTCGC3' | 5'GCCGCCATCCAGAGGGCAGA3' |
| IL6 | 5'CCTGAGAAAGGAGACATGTAACAAGA3' | 5'GGAAGGTTCAGGTTGTTTTCTGC3' |
| IL10 | 5'GGGCACCCAGTCTGAGAACA3' | 5'GACAAGGCTTGGCAACCCAG3' |
| MCP1 | 5'GCTCGCTCAGCCAGATGCAA3' | 5'TCCTGAACCCACTTCTGCTTG3' |
| P16 | 5’CCAACGCACCGAATAGTTACG3’ | 5’GCGCTGCCCATCATCATG3’ |
| P21 | 5'AGGTGGACCTGGAGACTCTCAG3' | 5'AGGTGGACCTGGAGACTCTCAG3' |
| Panx1 | 5'GCTCTTTGCGATCCTCCTGTA3' | 5'TGCACGGTTGTAAACTTTGTCAA3' |
| STAT1 | 5'TGTATGCCATCCTCGAGAGC3' | 5'AGACATCCTGCCACCTTGTG3' |
| TNFα | 5'ATGGGCTACAGGCTTGTCACTC3' | 5'CTCTTCTGCCTGCTGCACTTTG3' |
